# Deep learning accelerates whole slide imaging for next-generation digital pathology applications

**DOI:** 10.1038/s41377-022-00999-y

**Published:** 2022-10-14

**Authors:** Yair Rivenson, Aydogan Ozcan

**Affiliations:** 1 Pictor Labs, Inc., Los Angeles, USA; 2 Electrical and Computer Engineering Department, University of California, Los Angeles, 90095 CA USA; 3 Bioengineering Department, University of California, Los Angeles, 90095 CA USA; 4 California NanoSystems Institute (CNSI), University of California, Los Angeles, 90095 CA USA; 5 Department of Surgery, David Geffen School of Medicine, University of California, Los Angeles, 90095 CA USA

**Keywords:** Imaging and sensing, Biophotonics

## Abstract

Deep learning demonstrates the ability to significantly increase the scanning speed of whole slide imaging in histology. This transformative solution can be used to further accelerate the adoption of digital pathology.

Digital Pathology is a sub-field in pathology that considers the processes of image acquisition, management, distribution, annotation, and computer-aided analysis. Digital Pathology is rapidly growing and has already created a highly active academic and industry ecosystem^1^. The ability to digitize large scales of pathology data and rapidly disseminate and analyze them is transformative for research, clinical trials, telemedicine, downstream image analysis and overall patient care^2^. At the heart of this revolution, high-throughput scanning microscopes provide the “crude oil” that ultimately fuels this digital transformation. Some of these scanning microscopes and their digital images were approved by regulatory authorities to be used by pathologists in their primary diagnosis clinical workflow^3^.

It is undeniable that the next Machine Learning (ML) revolution in histopathology will be fueled by large-scale annotated databases. However, one of the biggest culprits is the current speed and throughput of digital pathology scanners^4^. Scanning each histology slide (using e.g., 40×/0.75NA objective lens) often yields Gigapixel-worth of information. While hardware and software advances in recent years resulted in a speed-up of the scanning time^2,4^, it is still a rate-limiting step that adds a burden on innovation in the field, as the price of these high-throughput scanners is in the range of ~$150–300 K, making the scanner a “unicorn” in many deployment scenarios, as purchasing multiple scanners becomes highly costly.

For a typical brightfield scanning microscope, the scanning speed rate-limiting step is often a result of the mechanical specifications of the scanning stage^2^. Many efforts have been put forward to increase the overall scanning speed of these microscopes. One solution involves adding hardware components to the microscope apparatus, such as coded illumination^5–8^, which then requires a postprocessing computational step to decode the encoded specimen information^9^. More recently, machine learning (ML) algorithms were also used to accelerate the throughput of digital microscopic scanning, including the enhancement of the resolution, depth of field^10^ and refocusing capabilities^11,12^, as well as the ability to computationally generate multiple stains on the same tissue section, effectively accelerating the imaging process by multiplying the useful information channels from each slide^13,14^. Another technology that a few vendors have adopted is based on Time Delayed Integration (TDI), which uses high-throughput scanning by synchronizing the charge transfer with the sample’s movement^15^. However, most of the existing slide scanners still rely on standard CCD/CMOS cameras without TDI capability.

In this issue of *Light: Science & Applications*, Michael John Fanous and Gabriel Popescu at the University of Illinois at Urbana Champaign (UIUC) report on a new method to significantly speed up the scanning speed of whole slide scanners^16^. It does so by departing from the standard “stop-and-stare” imaging approach to a continuous scanning approach, reconstructing the resulting image by applying a machine learning-based image restoration approach to the motion-blurred scanned image. This ML-based approach is implemented on a standard scanning microscope and eliminates the need for specialized add-on hardware components. The main contribution of this approach is decoupling the imaging speed from the stage movement speed and stage stabilization. To achieve their goal, the authors adopted the Pix2Pix image translation framework^17^. In this case, the input is a highly motion-blurred image, created by the fast movement of the stage, while the label is the sharp, high-resolution image captured at normal speeds. This approach has two key components. The first is *supervised* image-to-image translation^18^, which uses paired, accurately registered images to facilitate pixel-level optimization. The second component is a Generative Adversarial Network (GAN)^19^ based model used to craft a data-adaptive optimization. In a nutshell, GANs are composed of two competing networks: A Generator and a Discriminator. The role of the Generator is to take an input image (in this case, a substantially blurred one due to rapid motion) and to output an image that resembles the paired label, while the Discriminator’s role is to serve as a critique of the Generator’s output. This tug-of-war between the Generator and Discriminator pushes the Generator to match the labels’ sample distribution, which creates sharp and realistic-looking results. The fact that paired images are used in the Generator network allows the authors to add an L1 pixel-based loss term, that reigns the more “artistic” effects of the Discriminator and creates a highly accurate image reconstruction, opposed to images that only attempt to create the global distribution of the image^18^.

Following the training of the deep neural network, it was used to deblur images that were continuously scanned at stage speeds of up to 5000 µm/s with an acquisition rate of 30 fps equivalent to ~1.8 GPixels in 100 s, or an approximate area of 15 mm × 7.5 mm imaged with a standard high-throughput scanning microscope. For the sake of comparison, the ground truth images were captured at a stage speed of 50 µm/s—i.e., 100-fold slower (see Fig. 1).

**Fig. 1:**
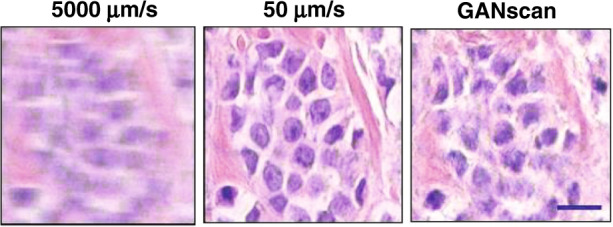
A demonstration of motion deblurring by GANscan. The results closely resemble the control specimen, with 100x times the acquisition speed

The authors demonstrated this for both brightfield and phase contrast microscopy applications and for multiple types of biopsies and tissue constituents. Moreover, authors have shown impressive resilience for slightly defocused imaging of +/−5 μm. This is important for any potential practical use of this approach, as defocusing is a common problem for these scanners (especially at high magnifications) and with the increasing number of focus correction steps, the substantial speed gain achieved by this all-algorithmic method would have been mostly lost.

This approach can benefit clinical and research workflows and increase the adoption of digital pathology by increasing the throughput of the scanning microscopes. It can utilize already deployed microscopes in clinical settings without additional hardware modifications. For this method to become widely adopted, the ML models should be fed with diverse training data, to cover specimens from multiple origins and microscopes. Overall, the method can help democratize research and power next-generation computer-aided diagnosis applications.

Through this News & Views article, we also would like to remember the senior author of this work, Dr. Gabriel (Gabi) Popescu, who passed away earlier this year in a tragic accident in Europe. He was a pioneer in holography, microscopy and quantitative phase imaging (QPI), and a role model and a wonderful colleague. Starting with his postdoctoral studies at MIT, Gabi focused on developing new technologies for label-free imaging of live specimens. He combined the strengths of optical microscopy, holography, and light scattering to reveal the intrinsic signatures of living cells without perturbing their natural state. While at UIUC, Gabi co-authored a number of key papers and patents, which were also commercialized by a start-up that he co-founded. As a continuation of his impactful research, his work on Spatial Light Interference Microscopy (SLIM) provided unprecedented sensitivity to cell structure and dynamics at the sub-nanometer scale, quantified cell growth with femtogram accuracy, and also detected cancer in unstained biopsies. White-Light Diffraction Tomography (WDT) is another technique he pioneered that uses SLIM data to extract cellular information in 3D. WDT taught us new science about how cells function in time and, because imaging is not perturbing the cells, the specimens could be investigated over longer periods of time.

Gradient Light Interference Microscopy (GLIM) was another relatively recent invention of Gabi that extended his techniques to thicker specimens, such as brain slices, embryos, and whole organism models (C. Elegans, zebrafish). GLIM is likely to revolutionize, for example, the understanding of neural connectivity in viable brain, without using labels or physical contact. In-vitro fertilization is another good match for GLIM, as typical contrast agents cannot be used on developing embryos without introducing risks of viability. GLIM was also commercialized through his lab’s pioneering entrepreneurial efforts.

The optics community has lost a giant! We are all deeply saddened and shocked by Gabi’s unexpected death earlier this year. Our community will remember him through his pioneering research contributions to the biophotonics field in general, his successful entrepreneurship in translating the exceptional scholarly output of his research group into impactful products, his outstanding teaching, mentorship, excellent service to the optics & photonics community and kind friendship and witty humor.
